# Standardized Patients in Medical Education: A Review of the Literature

**DOI:** 10.7759/cureus.42027

**Published:** 2023-07-17

**Authors:** Octavia L Flanagan, Kristina M Cummings

**Affiliations:** 1 College of Osteopathic Medicine, Lake Erie College of Osteopathic Medicine (LECOM), Elmira, USA; 2 Department of Family Medicine, Lake Erie College of Osteopathic Medicine (LECOM), Elmira, USA

**Keywords:** self-efficacy, student confidence, clinical competence, simulated live patients, medical school education, standardized patients

## Abstract

The concept of standardized patients (SPs) was first introduced in the 1960s by Dr. Howard Barrows of the University of Southern California and has been applied in medical school education since that time. This practice has allowed medical students to practice skills on live persons who are teachers rather than on real patients, who may be endangered by their emerging skills. Previous studies supported the use of SPs but did not measure whether they improved clinical competence or students’ confidence in their skills. This literature review evaluated whether current medical education literature supports or refutes the use of SPs compared to other modalities such as simulated patients (SiPs) and virtual reality (VR) in the improvement of student confidence, clinical performance, and interpersonal communication skills. The research questions posed for this review were as follows: do medical students in their first two years of education who have practiced skills using SPs have more self-confidence in their ability to perform skills on real patients than those students who did not use SPs, do medical students in their third and fourth years of medical school have higher clinical competency with sensitive patient examinations after using SPs in their first two years of medical education than those students who did not use SPs, and do medical students who have used SPs for discussing sensitive issues have better interpersonal skills when they encounter real patients in the clinical setting than those who have not used SPs?

The methodology for this descriptive, systematic review of the literature was organized using a Preferred Reporting Items for Systematic Reviews and Meta-Analyses (PRISMA) flowchart to describe how articles were collected and synthesized to evaluate the variables under study. The results of this study revealed that students learned the most when SPs were used because they were able to teach students the skills that they needed in a safe learning environment. Medical students performing sensitive patient examinations with SPs learned not only how to perform the examinations but also how to improve their communication with patients. Students and residents reported increased confidence and clinical competence when performing new skills with SPs rather than with peer practice, virtual reality, or real patients in a clinical setting. Although the utilization of SPs has been studied in multiple ways and found to be a powerful tool in the education of undergraduate medical students and interns, there is still much study to be done to address the human needs of real patients. Gaps in this literature included small sample sizes, a lack of standardized assessment tools, and the need to include a multidisciplinary approach that addresses cultural awareness and appreciation. The authors found limited studies analyzing the effect the coronavirus disease 2019 (COVID-19) pandemic had on the use of SPs in medical school education. Continued scientific inquiry in post-pandemic medical education is an essential component for dissemination as most schools have reintroduced the use of SPs, which strengthens the concept that their use is superior to the other simulation methods used when SPs were not available.

## Introduction and background

The concept of standardized patients (SPs) was introduced in 1963 by a medical educator, Dr. Howard Barrows, of the University of Southern California, Los Angeles. Dr. Barrows found that medical students expressed the need for an opportunity to practice medical skills prior to having to perform them on live patients. Dr. Barrows referred to those first SPs as “programmed patients,” and they have been called by a variety of names since, including patient instructor, patient educator, professional patient, and the more generic term, “simulated or standardized patients.” All these terms refer to a person who has been carefully trained to take on the characteristics of a real patient, or other person, to provide an opportunity for a student to learn or be evaluated on skills firsthand [1].

“While working with the standardized patient, the student can experience and practice clinical medicine without jeopardizing the health or welfare of real patients. It takes the process of learning a step beyond the books and away from reliance on paper and pencil tests. It allows the learner to have an encounter with a living, breathing, responding human being” [2].

Eight out of 195 accredited medical school campuses in the United States (medical doctor (MD) and doctor of osteopathic medicine (DO)) do not advertise the use of standardized patients in their online marketing and web pages [3,4].

In this literature review, the authors will show that the body of literature regarding the use of SPs is overwhelmingly supportive of their use in medical school over the course of a student’s education, starting in the first year and extending into residency. The authors found that students expressed increased confidence and less anxiety, demonstrated a higher level of clinical competency, and increased their interpersonal communication skills, after following a medical school curriculum that utilized SPs.

Bokken et al. (2010) studied how well 163 first-year medical students performed with live patients versus SPs in clinical encounters exploring communication skills. The students reported that SPs provided better feedback than live patients to assist the medical students in learning medical communication concepts [5,6]. Davies et al. (2015) reported that clinical performance and student self-confidence were positively correlated to the use of SPs, as evidenced by increased scores on clinical evaluations and self-reported confidence scores [7]. SP effectiveness was evaluated by Fortin et al. (2002) in a qualitative study with 91 first-year and 36 second-year medical students. The students were questioned on their perceptions of the encounters with the SP. Students reported that the SPs contributed to their learning of communication skills and were helpful in providing knowledge on how to best communicate with patients regarding psychosocial encounters. They reported that the use of the SPs helped them have more confidence in their interpersonal communication [8]. Another study completed by Bokken et al. (2010) utilized nine adolescent SPs, in adolescent scenarios with 341 medical students. The purpose of the study was to see if adolescents as SPs could provide meaningful feedback to medical students. Results found from interviews with the medical students revealed that most students found the experience beneficial and helpful using age-appropriate SPs in adolescent case study scenarios, and the adolescent SPs suffered no ill effects [6].

To better understand the role of SPs versus learning from colleagues, Power and Center (2010) looked at peers performing sensitive examinations on each other. The ones that returned the survey stated “this was the worst experience they had during medical school” and showed the need for an SP curriculum to practice skills for genital and breast examinations [9].

In a landmark study, Plauché et al. (1985) studied the effect of using SPs for sensitive patient examinations, particularly gynecologic examinations. This study showed that third-year medical students and resident physicians would rather use gynecologic teaching associates (GTAs) to learn and practice skills because they felt more comfortable and better prepared when they were required to see live patients. This study supported the need for standardized sensitive patient examinations, especially for those students who were not going to go into obstetrics and gynecology [10].

The purpose of this literature review was to look at the body of research and explore the value or detriment of utilizing SPs in medical school education and the effect SP use has on student self-confidence, clinical competence, and interpersonal communication skills in medical students and resident physicians. Additionally, the use of virtual reality and mannequin simulation was also considered. The authors of this paper wanted to determine whether the use of virtual reality and mannequin simulation could replace the use of SPs or if they each served an independent, adjunctive purpose in the student’s medical education.

Additionally, an inquiry was made into the traditional role of “real patients” being the primary source of student clinical practice in medical education or whether using SPs in the early medical education of the novice clinician would result in higher levels of clinical competence, interpersonal communication skills, less anxiety, and more self-confidence in these students.

Over the course of looking at the literature, several questions emerged: do medical students in their first two years of education who have practiced skills using SPs have more self-confidence in their ability to perform skills on real patients than those students who did not use SPs, do medical students in their third and fourth years of medical school have higher clinical competency with sensitive patient examinations after using SPs in their first two years of medical education than those students who did not use SPs, and do medical students who have used SPs for discussing sensitive issues have better interpersonal skills when they encounter real patients in the clinical setting than those who have not used SPs?

## Review

Methods

The aim of this literature review was to evaluate the current body of knowledge on the use of SPs in medical school education. The research questions served to evaluate whether the literature supports or refutes that student confidence, clinical competence, and interpersonal communication skills are more positively related to the use of SPs on these variables than the use of other patient simulation modalities, including real patients, in clinical practice.

To organize the results of the study, the authors grouped these data into the variables under study, which were those included in the research questions for this literature review: variable 1 was student self-confidence in the performance of skills, variable 2 was the performance of clinical competence by evaluation, and variable 3 was the use of interpersonal communication skills to include sensitive and difficult topics with patients and their families.

The methodology for this descriptive, systematic review of the literature was organized chronologically in the literature table to explore trends in the use and benefits of SPs in medical education. The time periods between 2005 and 2010, and between 2014 and 2018 revealed a surge in the literature regarding the use of SPs. When the coronavirus disease 2019 (COVID-19) pandemic was emerging and peaking, between the years 2020 and 2022, there were few articles specific to the use of SPs because their use was discontinued during this time of limited face-to-face educational instruction.

Articles were collected with the use of commonly used medical databases such as PubMed Central, EBSCOhost, Cumulative Index to Nursing and Allied Health Literature (CINAHL), Full Text Finder from Lake Erie College of Osteopathic Medicine electronic journal holdings, Medline, and PsycINFO.

The successful keywords selected to obtain articles that were applicable to our research questions and variables under study included “standardized patients,” “medical school education,” “clinical competence,” “interpersonal communication,” and “student confidence” or “self-efficacy.”

To exclude studies that did not fit the research questions of the study, the inclusion criteria were articles written in English, date ranges from 2008 to 2023 (no older than 15 years from publication), and landmark articles for years prior to 2008. Additionally, the literature included from intervention and review articles had to include the use of live SPs during the four years of medical school education or during medical residency and explored the variables under study, as delineated above.

Using these criteria, we finalized 40 articles in total. Of those articles, we had 23 intervention studies, 16 review papers, and one case study review. Within the review articles that were cited, seven of these also had descriptive or informational data related to the use of SPs, which was thought to help the novice reader understand the concept of the use of SPs and the variables under study more clearly.

The variables under study were represented in the 40 articles as follows: variable of student confidence, self-efficacy, or reduction of anxiety (12 articles), variable of clinical competence ratings of students by either SPs or faculty or by student report (15 articles), variable of interpersonal communication skills (20 articles), and variable of problem-based learning (PBL) medical school preparation to clinical (five articles).

**Figure 1:**
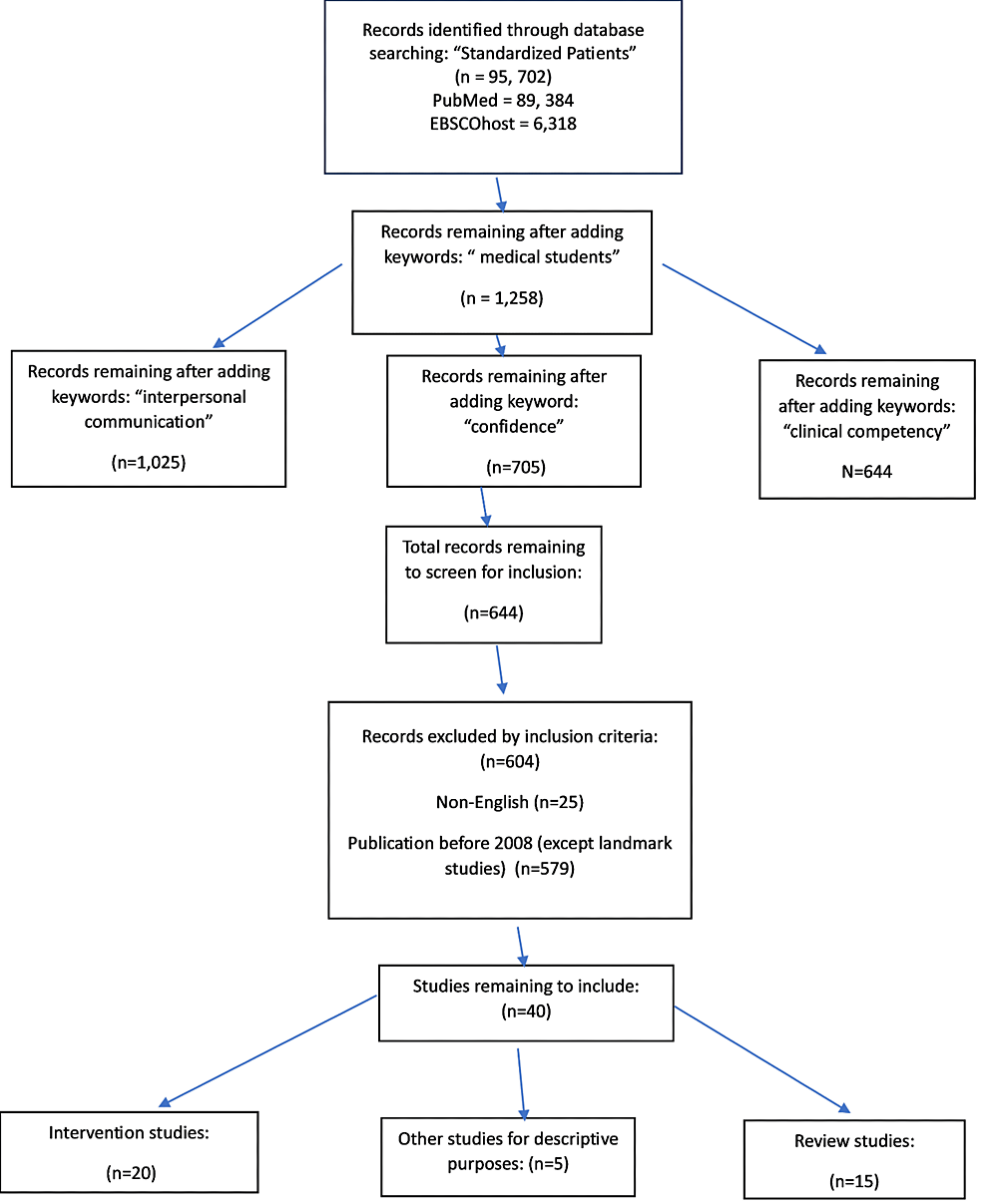
PRISMA systematic literature review N: number of articles selected, PRISMA: Preferred Reporting Items for Systematic Reviews and Meta-Analyses PubMed and EBSCOhost are literature databases for medicine and health sciences.

The total represented above is over 40 because many of the articles included more than one variable being examined per article. These data are organized into the Preferred Reporting Items for Systematic Reviews and Meta-Analyses (PRISMA) flow diagram presented in Figure 1.

Results

Fifteen articles in this review addressed the variable of clinical competence after using SPs in the four years of medical school. Twelve were focused on student confidence, or self-efficacy, in the ability to perform clinical skills for the breast and gynecologic portions of the examination for females and the genitourinary portions of the examination for males. Self-efficacy and student confidence were also measured and analyzed for communication skills for sensitive and difficult patient interactions. Twenty articles were reviewed to explore interpersonal communication skills using SP encounters. Another seven articles explained the nature of SPs, as well as the gaps in the educational literature on the use of SPs.

Student Self-Confidence

In a landmark study by Plauché et al. (1985), 420 third-year medical students were evaluated for clinical competence and rated for student self-confidence in an intervention using GTAs. The highest-ranked answers from the questionnaire were for increased student comfort, a successful learning experience, and an increase in knowledge. Qualitative data from this study included quotes from students in which graduates of the program felt they would benefit from using GTAs to practice throughout the curriculum. Most of the students in this study strongly disagreed that the use of GTAs was a “waste of time” [10].

Similarly, Beckmann et al. (1986) studied student response to GTAs in 292 second- and third-year medical students and found that almost 100% of the sample reported GTAs to be “outstanding and helped to alleviate anxiety” [11].

In a large 10-year systematic literature review between 1996 and 2005, May et al. (2009) reviewed 866 articles that studied the use of SPs in graduate nursing student education. They found that students reported positive outcomes in knowledge skills and attitudes after having had experiences with SPs prior to seeing real patients in the outpatient setting. Medical students and graduate nursing students have the same type of clinical experience, so results would be appropriate for this review [12].

Student Clinical Competence

Other major studies include interventions aimed at the use of SPs to improve clinical competence. McGraw and O’Connor (1999) performed a quasi-experimental intervention using 75 first-year medical students and found that using SPs helped student learning when utilized in small student groups of four to six early in their medical school education. Additionally, the study supported the conclusion that student clinical competence was not adversely affected when not experiencing real patient encounters [13].

Wånggren et al. (2005) conducted a landmark intervention study to look at the use of GTAs in medical school education, evaluating student skills and feelings. All 48 medical students reported that they felt they were more competent at performing sensitive physical examinations using GTAs. The GTAs were also able to provide appropriate communication to students while performing this sensitive physical assessment to guide them to the correct technique. Additionally, the students highly rated the ability to receive immediate feedback from the SPs in this way. The SP, in this case, was acting in the role of teacher and patient [14].

SPs are commonly used with students to assess their clinical competence. Epstein and Hundert (2002) did a landmark systematic literature review, in which they chose 195 relevant articles and explored professional competence in medical students’ assessment skills, interpersonal communication, and professionalism. The results of this study showed a need to standardize the forms used to evaluate clinical competence in medical students, as well as the need to develop a multidisciplinary assessment approach [15].

Theroux and Pearce (2006) completed an intervention study with 28 graduate medical and nursing students, measuring the competence and comfort level they had in performing pelvic examinations after working with SPs. This qualitative, longitudinal study was performed over three years in school and found that those students who utilized SPs expressed more self-efficacy than those who practiced on their peers [16].

More recently, Cifuni et al. (2020) performed an intervention study on first-year emergency medicine interns transitioning to medical practice. The authors wanted to see what the interns felt about using SPs prior to seeing real patients. Of the interns, 90% reported that this experience would change their clinical practice positively. After one year, 75% of residents reported that their experiences with the SPs did in fact change their clinical practice and that they established good habits early in their internship year [17].

Interpersonal Communication Skills

Spencer et al. (2000) explored patient-oriented learning environments with the use of SPs in a landmark study. They focused on the role of standardized patients as teachers in educating medical students on communication skills, empathy, and professional attitudes. This literature review found that using SPs as teachers provided context to their history and physical course content and allowed faculty to evaluate how essential the role of SPs was in the development of strong student interpersonal communication skills [18].

Kneebone et al. (2006) along with several other studies included in this review looked at the use of SPs in contrast to simulation using virtual reality and other simulation methods, such as mannequins and simulators. The overarching conclusion from these studies is that humans, in the form of SPs, can trigger authentic responses from the students that the other modalities do not elicit [19-22].

Bokken et al. (2010) used adolescent SPs for teaching communication skills to medical students in their first two years of study; 341 students participated in the study and felt that their communication skills improved due to the use of age-appropriate SPs [6].

Block et al. (2018) completed a systematic literature review and found that students highly rated the feedback they received from SPs. These students expressed that working with SPs improved their communication skills, and they expressed the desire to work with the same SPs to learn communication skills in scenarios over time that supported the concept of continuity of care [23].

PBL and Communication Skills

Diemers et al. (2007) completed a qualitative intervention study with 24 medical students in their third year of a six-year medical education program, which was their first year in medical school. The purpose of the study was to evaluate clinical skills after doing case-based learning scenarios in class using live SPs. The data collected from three focus groups revealed that the use of SP encounters was very helpful in solidifying concepts they learned in the case [24]. The following year, Diemers et al. (2008) published a second study in which they studied students in the problem-based learning (PBL) track from the Netherlands in their first and second years of medical school. They found that SP encounters reinforced communication skills learned in PBL. The students felt that experiential learning utilizing SPs increased their communication, medical knowledge, and clinical skills [25].

Lane and Rollnick (2007) did a systematic literature review, starting with 5,305 references, to study the use of SPs in PBL role-play and compare those to the groups that did not use SPs. The students in these studies more often would report that their communication skills were more improved in the sessions utilizing the SPs [26].

Yoon et al. (2016) studied 99 medical students who were enrolled in a PBL educational curriculum looking at the use of SPs in PBL role-play. They compared role-play using SPs versus video encounters, and most medical students evaluated sessions more positively with the use of standardized patients versus videos [22].

Table 1 presents the entirety of the results of this study [5-42].

**Table 1:** Findings of the literature review Systematic research of all the literature led to the inclusion of 40 relevant studies to address the research questions outlined in the first section of this review paper. CE, continuing education; GTA, gynecologic teaching associate; LGBTQ, Lesbian, Gay, Bisexual, Transgender, and Queer, MS, medical student; PBL, problem-based learning; PE, physical examination; SP, standardized patient; VR, virtual reality

Year	Author(s)	Article title	Subjects	Methodology	Variables/themes	Findings/conclusion
1985	Plauché and Baugniet-Nebrija [10]	Students’ and physicians’ evaluations of gynecologic teaching associate program	420 third-year medical students	Intervention, GTAs, landmark study	Clinical competence and student confidence	The highest mean rank answers were for increased student comfort, well-prepared GTAs, successful learning experience, and knowledge increased.
1986	Beckmann et al. [11]	Student response to gynecologic teaching associates	292 respondents (173 MS2 and 119 MS3)	Intervention, GTAs, landmark study	Student confidence	99% of samples from MS3 found GTA to be excellent/outstanding for learning skills; 98% (n=173) of MS2 found GTA to be outstanding and alleviate anxiety.
1999	McGraw and O’Conner [13]	Standardized patients in the early acquisition of clinical skills	75 first-year MS	Intervention: quasi-experimental	Clinical competence	The results show that the SP model was well received by students and that not working directly with real patients initially did not adversely affect learning. Gaps: Not randomly assigned to groups so students could choose to be in the treatment group, which is a design flaw.
2000	Spencer et al. [18]	Patient-oriented learning: a review of the role of the patient in the education of medical students	39 articles	Literature review	Communication skills	Review of the Cambridge model, which trains former patients to become simulated patients for medical education; it has been shown to be effective in teaching communication skills to first- and second-year medical students.
2002	Epstein and Hundert [15]	Defining and assessing professional competence	195 relevant articles chosen	Literature review	Clinical competence	The aim of this study was to explore the literature for a standardized instrument to measure medical student assessment skills, interpersonal communication, and professionalism. The study showed the need for the creation of a multidisciplinary assessment.
2002	Fortin et al. [8]	Teaching pre-clinical medical students an integrated approach to medical interviewing: half-day workshops using actors	91 first-year students and 36 second-year students	Intervention	PBL curriculum and communication skills	Compared actors as SPs and role-play in PBL; students said they liked actors/SPs because they were not afraid to hurt a patient.
2005	Wånggren et al. [14]	Teaching medical students gynaecological examination using professional patients-evaluation of students’ skills and feelings	48 medical students, 51 faculty members, and 53 SPs completed questionnaires	Intervention	Clinical competence and communication skills	Students, teachers, and SPs found this type of medical education to be of great value. Students reported less stress and anxiety; after training with SPs, students reported feeling relieved and calmer than when working with “real” patients.
2006	Kneebone et al. [19]	The human face of simulation: patient-focused simulation training	Descriptive paper not applicable	Other: descriptive article for information	Student confidence and communication skills; SP superior to VR	It was found that SPs were rated higher than VR. The study concludes that using real humans for simulation (SPs) of surgical skills can trigger authentic responses from trainees on a level that computers or models cannot do.
2006	Theroux and Pearce [16]	Graduate students' experiences with standardized patients as adjuncts for teaching pelvic examinations	28 graduate (medical or nursing) students	Qualitative method, intervention	Student confidence and comfort level	Longitudinal over three years in school; compared peer PE practice with SP; SP is more effective in improving self-efficacy.
2007	Diemers et al. [24]	Students’ perceptions of early patient encounters in a PBL curriculum: a first evaluation of the Maastricht experience	24 medical students in third year of a six-year medical education program in three focus groups	Intervention	Other: PBL to clinical practice	Evaluation of clinical skills after doing case-based PBL scenarios in class using live standardized patients; students report that encounters help solidify concepts learned in the case.
2007	Lane and Rollnick [26]	The use of simulated patients and role-play in communication skills training: a review of the literature to August 2005	5,305 references investigated	Literature review	PBL and communication skills	The authors looked at the use of simulated patients with role-play in PBL to those without SPs; the students more often would report their sessions more positively with the use of SPs than without.
2007	Linssen et al. [37]	Simulating the longitudinal doctor-patient relationship: experiences of simulated patients in successive consultations	23 third-year medical students	Intervention	Communication skills	Eight males and 15 females; the mean age of the entire group was 61. Experiences of SPs with third-year MS revealed better relationships with students working overtime with them; students provided feedback after each session; both MS and SP liked being with the same student throughout the practical experience of successive patient-doctor consultation.
2007	Rethans et al. [27]	Unannounced standardised patients in real practice: a systematic literature review	85 articles	Literature review	Clinical skill acquisition with the use of incognito SPs	Despite physicians not knowing the patient was an SP rather than a “real” patient, they reported more satisfaction when they were testing with SPs.
2008	Bokken et al. [28]	Strengths and weaknesses of simulated and real patients in the teaching of skills to medical students: a review	Did not report the number of articles, PubMed and Eric databases	Literature review	Clinical competency and student confidence	This literature review examined the strengths and weaknesses of the use of SPs in reducing anxiety in medical students and improving clinical competency. Discovered that the use of SP as teachers has been found to be highly valued and indispensable to *undergraduate* medical education, providing a safe, low-anxiety learning environment.
2008	Diemers et al. [25]	Students’ opinions about the effects of preclinical patient contacts on their learning	24 first- and second-year preclinical medical students from the Netherlands	Intervention study using focus groups; qualitative methods	Other: PBL to clinical practice	Using SPs in early medical education was found by students to be a way they can connect knowledge and clinical reasoning skills in PBL to live SP encounters and practice skills with less anxiety.
2009	Bokken et al. [40]	Students’ views on the use of real patients and simulated patients in undergraduate medical education	38 first- and second-year medical students	Qualitative methods, intervention study	Clinical competency and student confidence	Qualitative study designed to assess student’s attitudes on strengths and weaknesses of using SPs rather than “real” patients in an undergraduate medical curriculum. Discovered that students preferred SP interactions to be better prepared for real patient interactions. Students revealed that they felt more confident in their skills when they practiced with SPs.
2009	May et al. [12]	A ten-year review of the literature on the use of standardized patients in teaching and learning: 1996-2005	866 English language studies	Literature review using Kirkpatrick’s model	Student confidence	Most studies revealed that the educational use of SPs was valuable and discussed a need for further research.
2009	McGaghie et al. [20]	Lessons for continuing medical education from simulation research in undergraduate and graduate medical education: effectiveness of continuing medical education: American College of Chest Physicians Evidence-Based Educational Guidelines	Did not include the number of articles reviewed in this review	Literature review	Clinical competence and communication skills; SP encounters are superior to VR encounters	Supports the use of SPs for psychomotor skills, performing PE, and communications; medical simulation (such as VR) supports but does not replace experience with SP or real patients.
2010	Bokken et al. [5]	Instructiveness of real patients and simulated patients in undergraduate medical education: a randomized experiment	163 first-year medical students	Intervention study	Communication skills	The study showed that real patient encounters were not as helpful with practicing both communication skills or giving meaningful feedback to the medical student; SPs have been trained to be informed about what skills to evaluate and give meaningful feedback to students to help with learning concepts/skills.
2010	Bokken et al. [6]	The case of “Miss Jacobs”: adolescent simulated patients and the quality of their role playing, feedback, and personal impact	341 medical students using nine adolescent girls as SPs	Intervention	Communication skills	Students and teachers felt that role-playing and the feedback provided by adolescent SPs were valuable; no negative comments about performance.
2010	Power and Center [9]	Examining the medical student body: peer physical exams and genital, rectal, or breast exams	Fourth-year medical students; 138 completed surveys	Intervention using mixed method design	Student confidence and comfort level	The study focuses on the entire peer-to-peer examination, including breast, genital, or rectal examinations (GRB). The purpose of the study was to measure the discomfort of students performing skills on each other in the entirety of the examination and not focusing on the GRB examination (that not all students performed). Thirty-two students who did GRB provided qualitative data that described doing these examinations on peers as “inappropriate, terribly uncomfortable, and awful.”
2012	Marwan et al. [32]	Are medical students accepted by patients in teaching hospitals?	932 medical students	Intervention study	Clinical competence	The study was designed to look at how many “real” patients refused to have medical students perform their examination and surmised that SPs would be a valuable resource because students would all get the same opportunity for practice compared to those who were refused by real patients. Notable was that most refusals were made to students needing to perform a gynecologic examination. The study supports the use of GTAs for the practice of these skills.
2013	Colbert-Getz et al. [30]	How do gender and anxiety affect students’ self-assessment and actual performance on a high-stakes clinical skills examination?	202 rising fourth-year medical students	Other: gender differences in self-efficacy	Student confidence	Females underrate themselves, while males overrate themselves; the study was looking at gender and clinical performance.
2013	Coleman et al. [38]	Summit on medical school education in sexual health: report of an expert consultation	Report on the results of an educational summit	Other: medical school curriculum needs	Communication and skills	Not a study using SPs; sexual health curriculum needs to be integrated throughout medical training; multidisciplinary approach.
2014	Dabson et al. [29]	Medical students’ experiences learning intimate physical examination skills: a qualitative study	16 Australian students: in Y2-Y5 of medical school; 12 females/4 males	Other: results are meaningful in how students are stressed practicing intimate skills on patients or each other	Student confidence and anxiety using other learning modalities than SPs	Student discomfort with the experience of learning intimate physical examination skills may be common and has ongoing repercussions for students and patients. Also includes discussion about levels of discomfort and beliefs about how helpful this practice is to them.
2014	Knight et al. [39]	Examining clinicians’ experiences providing sexual health services for LGBTQ youth: considering social and structural determinants of health in clinical practice	24 clinicians: 5 physicians and 19 nurses	Other: application to communication skills	Communication	Qualitative study revealed that clinicians felt frustrated by the lack of clinical skills to treat LGBTQ patients, and participants felt that patients in this community should have either a specialist to care for them or clinicians need in-depth training to care for them.
2015	Davies et al. [7]	Changes in student performance and confidence with a standardized patient and standardized colleague interprofessional activity	29 pharmacy students	Intervention study	Clinical competence and student confidence	The results of the study reveal that student confidence was positively correlated with clinical competence; measures of student clinical performance and student attitudes were tested.
2015	Oh et al. [33]	The effects of simulation-based learning using standardized patients in nursing students: a meta-analysis	18 studies that included 1326 total subjects in undergraduate and graduate nursing programs	Literature review, meta-analysis; four randomized and 14 non-randomized studies included	Clinical competence and student confidence	The authors found that the use of SPs in graduate study of nursing had beneficial effects on students in self-efficacy, knowledge and skill acquisition, and improved motivation with improved clinical skills as evidenced by faculty evaluation. They found gaps in the literature related to studies of small sample sizes.
2016	Yoon et al. [22]	Using standardized patients versus video cases for representing clinical problems in problem-based learning	99 medical students in PBL curriculum	Intervention	Other: PBL to clinical practice	The authors looked at the use of standardized patients with role-play in PBL to those without SPs; the students more often would report their sessions more positively with the use of SPs than without.
2017	Kaplonyi et al. [35]	Understanding the impact of simulated patients on health care learners’ communication skills: a systematic review	Review included a total of 60 studies	Literature review	Communication skills	SP-based education is widely accepted as a valuable and effective means of teaching communication skills. Gaps and limitations include a lack of outcome collection methods.
2017	Sattler et al. [41]	Actual and standardized patient evaluations of medical students’ skills	27 medical students	Intervention study; mixed methods	Student confidence, clinical competence, and communication	Compared the results of medical student communication skills using data collected from actual patients versus standardized patients and found that students valued practical feedback from SPs. Also felt that SPs and APs complement each other.
2018	Block et al. [23]	Perceptions of a longitudinal standardized patient experience by standardized patients, medical students, and faculty	34 studies in the review	Qualitative methods, literature review	Communication	Themes from students’ comments emerged that focused on the need for timely feedback from faculty and SPs and that both student and SP preferred to have the same pairing throughout training.
2018	Ramey et al. [31]	Implementation of standardized patient program using local resources in Avalon School of Medicine	24 student volunteers	Quantitative intervention study, pre- and post-test design	Clinical competency	SPs increased overall clinical competency in medical students; also, SPs strengthened learning and SPs benefit patients by safeguarding them from the clinical incompetence of novice students.
2018	Wilbur et al. [42]	Systematic review of standardized patient use in continuing medical education	5 studies were used in the review, although 488 were reviewed	Systematic literature review	Clinical competence in professional medical education	The results highlight the need for rigorous study in continuing medical education for physicians that includes the use of SPs. Only 5/488 articles on the use of SPs in medical education were selected that looked at professional medical CE.
2020	Chung et al. [21]	Videotaped unannounced standardized patient encounters to evaluate interpersonal and communication skills in emergency medicine residents	16 medical residents each having completed four different case scenarios (total of 64 encounters)	Intervention study	Communication skills	The results from this study showed that the use of unannounced videotaped observations of medical residents working in emergency medicine was an effective tool to evaluate skills when working with standardized patients.
2021	Cifuni et al. [17]	A standardized patient experience: elevating interns to expected level of clinical competency	Not applicable	Intervention study	Clinical competency and communication skills	90% of interns reported that clinical experiences using SPs would change their clinical practice; the faculty felt that the learning experience allowed for the identification of problems early and provided early guidance. The authors suggested that these findings show that the use of SPs provides foundational experience for interns to establish good habits early in their internship.
2021	Papanagnou et al. [34]	Developing standardized patient-based cases for communication training: lessons learned from training residents to communicate diagnostic uncertainty	Descriptive paper not applicable	Other: introducing possible protocol for intervention	Communication skills	Introducing protocol for developing case studies using SPs; successful integration of SPs into communication skills training program must include a well-thought-out procedure for developing case studies.
2022	Jones et al. [36]	Antiviral pharmacology: a standardized patient case for preclinical medical students	Start of the study: a total of 189 preclinical pharmacy students in two groups; attrition at the end of the study: a total of 133 in two groups	Intervention study experimental design	Communication skills	The results revealed that the use of SPs early in the preclinical period of medical school education helped them integrate pharmacology into the encounter and students reported high satisfaction. The results highlight the value of interactive SP learning in the communication skills of preclinical education.

Discussion

The authors of this study reviewed both research intervention and literature review papers that explored the use of an SP curriculum for teaching both clinical skills and interpersonal communication skills in medical school education. The concepts under study included the variables of student self-confidence, ratings from faculty, SPs, and students themselves regarding clinical competence and interpersonal communication skills. This review concluded that the use of SPs prior to working with “real” patients resulted in more student self-confidence and a reduction of student anxiety in performing novice skills. Students demonstrated better clinical skills with sensitive patient examinations using SPs than those who did not use an SP curriculum.

Student Self-Confidence

The concept of using SPs as teachers was explored in this review of the literature. Rethans et al. (2007) [27] and Bokken et al. (2008) [28] reported that the students rated the use of SPs as teachers as highly valuable. They felt that SPs provided a safe learning environment where they could make mistakes and receive feedback, and they revised the method by which they completed the task. Since the SPs were trained, they were aware of what to expect for their examination and could feel free to tell the student the mistakes they had made. In turn, students reported that they could make a mistake without the repercussions of hurting a live patient. Dabson et al. (2014) reported that when students were able to practice the sensitive patient examination with a SP, they reported that they had less anxiety and discomfort because they would be better prepared to perform skills with a live patient and lessen the chance of making a mistake with them. Students also reported that they felt very distressed about having to perform intimate examination skills with their peers, and the use of SPs eliminated that anxiety [29].

Colbert-Getz et al. (2013) examined the attitudes of male and female medical students and their confidence in practicing and performing new skills. Their study revealed that male students tended to feel more confident and rate their performance higher than their female counterparts. This study was included in this review to highlight a possible learning issue that could be present in medical students. The need for further research on this phenomenon could guide medical educators to teach skills in which the student could demonstrate a high competence level, which in turn may increase confidence in some female students [30].

Student Clinical Competence

When looking at a student’s ability to perform clinical skills, it was most noted that they felt better prepared to see “live” patients after having utilized SPs to “practice” skills with. Students reported that they were better prepared and more competent for real patient interactions when they practiced their assessment skills with SPs before going into real clinical sessions. Resident physicians who encountered an unknown patient (not knowing whether they were standardized patients or real patients) preferred SPs when revealed, as the SPs acted as teachers, whereas real patients did not [27].

Ramey et al. (2018) showed that SPs increased overall clinical competency in medical students and strengthened their skills. Patients benefited from students who had completed their clinical skill learning with SPs by safeguarding them from the clinical incompetence of a “new” clinician [31]. Marwan et al. (2012) showed that actual clinic patients would refuse to have students perform sensitive patient examinations if the student did not display a level of confidence with this examination. The use of GTAs assured that all students were able to practice these skills regardless of patient refusal [32].

The use of SPs has been shown by this study to enhance the use of psychomotor skills and patient communication. When compared to virtual reality (VR), the use of SPs was seen to support, but not replace, the experience of performing skills with SPs [20]. Kneebone et al. (2006) found similar findings, concluding that “using real humans in an SP role can trigger more authentic responses from trainees on a level that computers and models cannot” [19]. Oh et al. (2015) explored graduate nursing students using SPs and revealed a beneficial effect on self-efficacy, knowledge and skill acquisition, and improved motivation with improved clinical skills in faculty evaluations. The examination skills taught in advanced physical assessment courses in graduate nursing education are comparable to those in medical school; therefore, this study is applicable to medical school education and SPs [33].

Interpersonal Communication Skills

Papanagnou et al. (2021), in one of the descriptive articles, discussed the need to develop protocols for the use of SPs regarding communication skills training in a PBL curriculum. The authors proposed that protocols must include a specific, well-thought-out procedure for the development of the case studies used [34]. Kaplonyi et al. (2017) also demonstrated that the use of SPs early in the curriculum for first- and second-year medical students helped them improve their communication skills [35]. Jones et al. (2022) also noted that the use of SPs early in preclinical education was helpful in integrating pharmacology and basic sciences [36].

Linssen et al. (2007) showcased that medical students in their third year of education who worked with the same SPs over time improved their communication skills more consistently than those who worked sporadically with a different SP each time [37]. Block et al. (2018) corroborated that students and SPs preferred to have the same pairing throughout medical school training as it helped students work on skills longitudinally [23].

Coleman et al. (2013) established that a multidisciplinary approach to sexual healthcare was warranted [38]. Knight et al. (2014) reported that clinicians felt frustrated because they lacked clinical skills specific to the medical approach with the assessment and management of patients from the Lesbian, Gay, Bisexual, Transgender, and Queer plus (LGBTQ+) community, and these clinicians felt that if they had the opportunity to learn and practice this specific skill set with properly trained SPs when they were in their medical education, they would be better prepared and more competent to work with these patients [39].

Bokken et al. (2009) [40] and Sattler et al. (2017) [41] successfully explored attitudes on the strengths and weaknesses of using SPs in practicing new skills rather than with “real patients.” They revealed that students placed greater value on the practical feedback from SP encounters and added that SPs and “real” patients can complement each other without excluding the other. Furthermore, Wilbur et al. (2018) highlighted the need for ongoing studies in medical education using SPs [42].

## Conclusions

The reality of medical practice in the United States is based on a productivity model that requires medical providers to see many patients each day to make up for the lack of reimbursement by insurance. This limits the time for teaching or practicing novice skills, making it challenging for medical students to master the decision-making process required for patient care. To overcome this obstacle, medical practice needs an alternative method to better prepare students for clinical training. This review highlighted the rich body of literature that demonstrates the effectiveness of the use of SPs in medical curricula. There is overwhelming evidence to show that using SPs in medical education substantially increases student confidence, clinical competence, and interpersonal communication skills. Moreover, it comprehensively prepares them for the actual clinical encounters on their clinical rotations and beyond. In contrast, there is a lack of meaningful studies on the effectiveness of VR versus SP curriculum and the cost-effectiveness of VR. Newer studies are needed to evaluate more innovative approaches to SP education, as much of the current literature is outdated. Small sample sizes limit the strength of the study conclusions, making it difficult to generalize the findings to other populations of medical students. Studies also lacked reliable instrumentation for measuring positive outcomes that quantify the effectiveness of using SPs. In a post-pandemic environment, it is crucial to study how COVID-19 affected medical students’ skills, confidence, and communication.
